# Immune Modulation Related to High-Dose Valacyclovir Administration for Primary Cytomegalovirus Infection in Pregnancy: An Insight into Virus Behavior and Maternal Serology

**DOI:** 10.3390/v17020157

**Published:** 2025-01-24

**Authors:** Marco De Santis, Silvio Tartaglia, Chiara Cerra, Daniela Visconti, Piero Valentini, Antonio Lanzone, Lucia Masini, Rosaria Santangelo

**Affiliations:** 1 Ostetricia e Patologia Ostetrica, Dipartimento di Scienze della Salute della Donna, Fondazione Policlinico Universitario A. Gemelli IRCCS, del Bambino e di Sanità Pubblica, 00168 Rome, Italy; marco.desantis1@policlinicogemelli.it (M.D.S.); daniela.visconti@policlinicogemelli.it (D.V.); antonio.lanzone@policlinicogemelli.it (A.L.); lucia.masini@unicatt.it (L.M.); 2 Istituto di Clinica Ostetrica e Ginecologica, Università Cattolica del Sacro Cuore, 00168 Rome, Italy; 3 Center for Fetal Care and High-Risk Pregnancy, Department of Obstetrics and Gynecology, University “G. D’Annunzio”, 66013 Chieti, Italy; chiara.cerra93@gmail.com; 4 Pediatria, Dipartimento di Scienze della Salute della Donna, Fondazione Policlinico Universitario A. Gemelli IRCCS, del Bambino e di Sanità Pubblica, 00168 Rome, Italy; piero.valentini@policlinicogemelli.it; 5 Istituto di Clinica Pediatrica, Università Cattolica del Sacro Cuore, 00168 Rome, Italy; 6 UOC di Microbiologia, Dipartimento di Scienze di Laboratorio e Ematologiche, Fondazione Policlinico Universitario A. Gemelli IRCCS, 00168 Rome, Italy; rosaria.santangelo@policlinicogemelli.it; 7 Dipartimento di Scienze Biotecnologiche di Base, Cliniche Intensivologiche e Perioperatorie, Università Cattolica del Sacro Cuore, 00168 Rome, Italy

**Keywords:** cytomegalovirus, valacyclovir, immune system, pregnancy, IgG avidity

## Abstract

Cytomegalovirus (CMV) infection during pregnancy poses significant maternal and fetal health risks. Valacyclovir, an antiviral drug, has been explored as a therapeutic option for managing primary CMV infections in pregnant women. This study investigates the effects of valacyclovir therapy on immune response maturation against CMV, maternal antibody levels, and viral replication during treatment. We conducted a retrospective observational study involving pregnant women diagnosed with primary CMV infection and presenting in utero infection who received high-dose valacyclovir therapy (8 g/day). A group started the therapy at diagnosis, while another group started only after positive amniocentesis. Maternal antibody levels (IgM, IgG, and IgG avidity) and PCR for CMV testing (in blood, urine, and saliva) were measured longitudinally during the second and third trimesters. Our findings indicate that early valacyclovir therapy is related to lower avidity levels over time and a delay in reaching a high IgG avidity level (18.22 ± 1.21 weeks) compared to the patients who started Valacyclovir during the second trimester after positive amniocentesis (14.52 ± 1.64 weeks; *p* = 0.066). The therapy does not condition the overall concentration of maternal CMV-specific IgM and IgG. While high-dose VCV does not directly target the mechanism of IgG avidity maturation, it can interfere with this process by reducing the viral load and antigen presentation, influencing IgG avidity maturation. Further research is needed to elucidate the long-term implications of potential immunological modulation induced by Valacyclovir and to optimize early diagnosis and the right treatment protocol during pregnancy.

## 1. Introduction

Cytomegalovirus (CMV) is the main cause of congenital infection worldwide, the most common cause of infection-related fetal malformation, and the leading cause of non-genetic sensorineural hearing loss [1]. There is neither a vaccine for this member of the herpesvirus family nor a specific intervention to prevent the transmission, so early diagnosis is crucial [2]. Since there is currently no definitive prenatal treatment to reduce vertical transmission or to cure the infection in utero, universal screening is not always offered to pregnant women [3]. The Italian guidelines have been recently updated and currently recommend a universal screening in line with other countries such as France, Canada, and Belgium [4,5].

However, the scenario has changed since Valacyclovir (VCV) has shown effectiveness in many clinical settings [6,7,8]. VCV is a prodrug of Acyclovir active against CMV DNA polymerase when administered at high doses (8 g/day). This therapy has been approved in Italy for treating fetal infection with mild-moderate symptoms since 2020 [6]. Antiviral therapy is not indicated in the case of asymptomatic fetuses despite the high risk of intrauterine infection. A recent consensus from the European Congenital Cytomegalovirus Initiative (ECCI) stated that VCV should be administered in cases with periconceptional or first-trimester maternal primary infection as early as possible after the diagnosis and until the result of the CMV PCR in amniocentesis (Grade A). The same experts suggest that in women with confirmed intrauterine infection, fetal treatment with high-dose VCV may be considered after discussion with an expert team (Grade C) [9].

Congenital CMV infection can occur both in primary and non-primary infection, most commonly in the first case. Prenatally, the confirmation of fetal infection relies on imaging (ultrasound and eventually magnetic resonance) [10,11,12] and, in the case of an absence of structural anomalies [13], amniocentesis to test the CMV genome presence in the amniotic fluid. The sensitivity of real-time PCR for the detection of CMV DNA in amniotic fluid is estimated at over 90% [14,15,16]. Amniocentesis is typically performed at least 6 weeks after the primary infection and after 20 weeks of gestation to assess intrauterine infection [15]. Additionally, CMV real-time PCR on urine, saliva, and blood samples from the mother is commonly utilized to detect the infection. The goal of prenatal screening is an early diagnosis of infection. Maternal serological testing for CMV involves analyzing specific IgG and IgM antibodies and IgG avidity [17]. In primary infections, IgM antibodies are produced within 1–2 weeks after infection and typically become undetectable within 6–9 months [18,19]. Specific IgG antibodies rise 1–2 weeks after IgM (2–4 weeks after infection), reaching high avidity within 3–6 months and remaining detectable for life [18]. The CMV-specific IgG avidity assay is regarded as a “gold standard” for dating the timing of an infection [19,20]. Avidity is defined as the overall aggregate strength of the interaction between a polyclonal immunoglobulin and its target antigen [21,22]. In CMV infections, IgG avidity peaks around 8 to 12 weeks after infection and can continue to mature gradually. Avidity maturation may be influenced by binding affinity, valency, and the structural arrangement of the antibody–antigen interaction [23]. Researchers have reported that congenital transmission of a primary CMV infection can be linked to a strong cell-mediated response to CMV, depending on IgG avidity [24]. In this context, antiviral therapy could potentially interfere with the cell-mediated immunological response and avidity maturation process. To date, no exhaustive evidence exists about the effects of VCV administration on the maternal immune response against CMV infection in pregnancy. This study aims to describe maternal immunological behavior in response to primary CMV infection during pregnancy and to evaluate any possible modification of anti-CMV-specific immunoglobulin maturation eventually induced by VCV therapy.

## 2. Materials and Methods

### 2.1. Study Design

This is a retrospective observational study reporting the serological maturation trends of pregnant women diagnosed with primary CMV infection during the first or second trimester. Our hospital has long been a referral center for infections in pregnancy. During the initial visit, all patients underwent a complete serological examination (IgM, IgG, and avidity IgG) and CMV real-time PCR testing on urine, saliva, and blood to confirm the diagnosis. Primary CMV infection was diagnosed based on a combination of CMV IgM and IgG positivity, low IgG avidity, or seroconversion of anti-CMV IgG in a previously seronegative individual woman. In the case of isolated positive IgM with negative IgG, complete serological and molecular testing was repeated after 2 weeks to ascertain eventual cases of false positivity.

For all patients diagnosed with CMV infection, amniocentesis was offered after 20 weeks of gestation and at least 6–8 weeks from possible infection, to assess the presence of viral replication in amniotic fluid in cases of suspected fetal infection. Inspired by the high-dose treatment recommended in previous studies [8], oral VCV therapy (Zelitrex^®^, GlaxoSmithKline; Brentford, UK) was administered at a dose of 8 g/day (2 g every 6 h) immediately following confirmation of CMV infection. This dosage has been associated with reducing major side effects [25,26].

Since 2016, pregnant patients referred to our institution for CMV infection have received high-dose VCV treatment after positive PCR for CMV on amniotic fluid as a compassionate therapy [7]. After the Italian recommendation update in 2020, antiviral therapy was started at diagnosis confirmation of primary infection in pregnancy. For those women, whose condition resulted in positive amniocentesis for CMV viral genome, high-dose VCV was continued until the delivery for the high risk of congenital infection. In patients with a negative amniocentesis, VCV was discontinued. Each month, all patients underwent a detailed ultrasound, a complete blood serology (IgM, IgG, and IgG avidity), and PCR testing to detect the viral genome in maternal blood, urine, and saliva until delivery. Liver and renal function were monitored at each examination to identify any potential adverse effects of VCV administration.

We retrospectively collected data about patients treated at our institution for primary CMV infection, presenting positive amniocentesis results but asymptomatic fetuses and treated with high-dose VCV therapy. Women with concurrent infections that affect the immune response, including those with a non-primary CMV infection, a negative amniocentesis or fetal symptoms detected via ultrasound, were excluded from this study. We compared the serology longitudinal trend, particularly regarding IgG avidity increasing during the weeks after the infection. To assess possible interference with the natural immune system maturation due to the VCV therapy, we compared the blood test results between those women who were treated with high-dose VCV from the confirmation of diagnosis until the delivery (Group A) to those who started the same treatment after positive real-time PCR testing of the amniotic fluid during the second trimester of pregnancy until the delivery.

### 2.2. Laboratory Test

Anti-CMV antibodies (IgG and IgM) were evaluated using a commercial kit (CVG CMV IgG and CMM CMV IgM IMMULITE^®^ 2000 Immunoassay Systems Siemens Healthcare Diagnostics Products Ltd., Glyn Rhonwy, Llanberis Gwynedd, LL55 4EL, UK). The results were interpreted as suggested by the manufacturer: in particular, a cutoff index (S/CO) ≥ 1.1 was considered positive, <0.9 was considered negative, and in the range 0.9–1.1 was considered questionable. IgG avidity was determined using a commercial kit (LIAISON ^®^ CMVIgG Avidity II, DiaSorin, Saluggia, Vercelli, Italy). IgG avidity was measured in a Relative Light Unit (RLU), a unit of measurement used to quantify the intensity of light produced in luminescence-based assays. A value <0.15 RLU defines a low IgG avidity; between 0.15 and 0.25 RLU is considered moderate, while values >0.25 have been considered diagnostic of a high avidity. CMV DNA was extracted from aliquots of 0.4 mL of each sample (blood, amniotic fluid, urine, and saliva in UTMTM viral transport media) by the QIAsymphony RGQ complete automated system (QIAGEN GmbH, Hilden, Germany). An Artus CMV QS-RGQ assay (QIAGEN GmbH, Hilden, Germany) combined with real-time PCR was performed on the samples. For amniotic fluid, urine, and saliva, the analytical sensitivity of the Artus CMV QS-RGQ assay is 42.5 copies/mL considering the purification and assay setup using the QIAsymphony RGQ system. For whole blood, 122 IU/mL was used as the limit of detection.

### 2.3. Data Collection

Data were extracted from medical records, including demographic information, obstetric history, CMV diagnostic tests, therapy regimen, and follow-up results. The following parameters were specifically recorded:

- Baseline characteristics: Maternal age, parity, gestational age at diagnosis, the timing of infection, and eventual maternal symptoms of infection.
- Serological markers: CMV-specific IgM, IgG titers, and IgG avidity index, measured at baseline and at regular intervals (every 4 weeks).
- Virological monitoring: CMV DNA levels in blood, urine or saliva assessed via PCR.
- Therapy details: VCV start time, therapy duration.

### 2.4. Statistical Analysis

Statistical analysis was performed using SPSS Version 20 (Statistical Package for Social Science, Chicago, IL, USA). The normality of data distribution was assessed using the Kolmogorov–Smirnov test. Categorical variables were expressed as frequencies, while continuous variables normally distributed were disclosed as mean ± standard deviation (SD). The means were compared using the Student’s *t*-test, whereas the chi-square and Fisher’s exact tests were employed to compare the frequencies between the two groups. A *p* < 0.05 was considered statistically significant.

## 3. Results

From 2016 to 2024, a total of n = 73 patients were diagnosed with CMV infection during pregnancy and were treated with high-dose VCV. Ten patients were diagnosed with a non-primary infection. Three cases of primary infection presented fetal signs of infection at the ultrasound, so were excluded from the present study. At amniocentesis, n = 38 women presented the intrauterine presence of the virus and continued the treatment until the delivery, while n = 22 patients with negative amniotic fluid stopped the therapy administration. Among the 38 patients presenting CMV DNA in amniotic fluid, n = 8 (Group A) patients started the VCV immediately after the serological confirmation of primary infection, while n = 30 pregnant patients (Group B) started antiviral therapy after the positive amniocentesis. The characteristics of the population are shown in Table 1.

Among patients with primary infection, there were no statistically significant differences in terms of baseline characteristics comparing the two groups. There were no differences in terms of timing of infection and diagnosis, initial viral localization, or time to PCR negativity in blood, saliva, and urine. Evidently, the two groups significantly differed, considering the timing and duration of the therapy.

**Figure 1:**
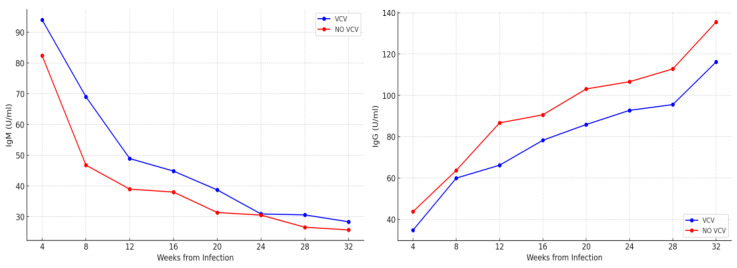
Patterns of specific anti-CMV IgM (**left**) and IgG (**right**) titers progress during pregnancy between the women undergoing VCV from diagnosis until delivery (Group A) and those who started the therapy after positive amniocentesis in the second trimester of pregnancy (Group B).

We then analyzed the maturation of the maternal immune response since the diagnosis was confirmed, evaluating serology patterns over time following primary CMV infection in all patients. Graphically, the patterns of IgM and IgG (Figure 1) titers progress during pregnancy describe a similar trend in the group of women undergoing VCV from the serological confirmation of infection (Group A) and those who started the therapy after positive amniocentesis in the second trimester of pregnancy (Group B).

**Figure 2:**
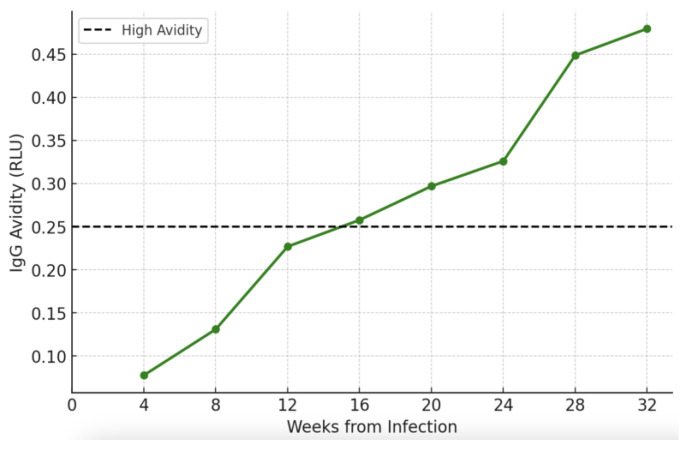
IgG avidity maturation trend in the population of pregnant patients presenting primary CMV infection undergoing high-dose VCV therapy.

The IgG avidity maturation trend (Figure 2) in pregnant patients with primary CMV infection and positive amniocentesis (n = 38), described a curve comparable to previous findings [21]. Our population’s mean time to reach IgG high avidity was 15.02 (±1.67) weeks.

**Figure 3:**
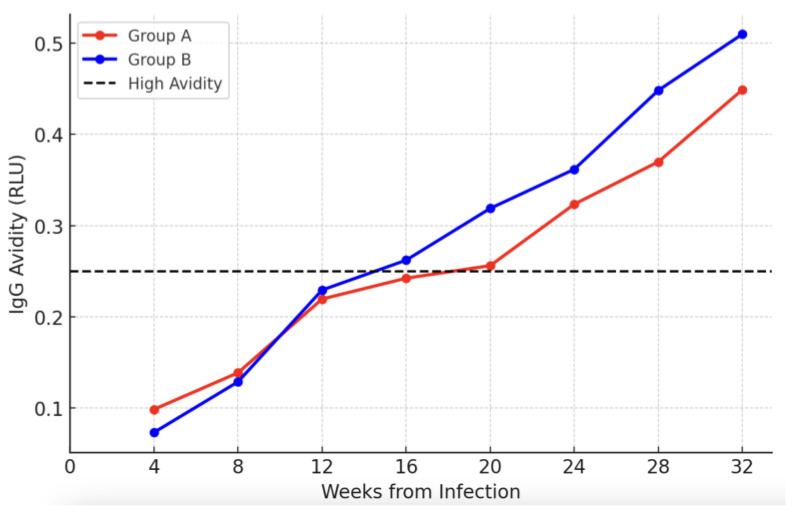
IgG avidity progress between women with a positive PCR for CMV DNA in amniotic fluid treated with VCV from the diagnosis of primary CMV infection throughout the pregnancy (Group A, in red) and women who started the therapy after positive amniocentesis (Group B, in blue).

We compared the progress in IgG avidity between women treated with VCV throughout the pregnancy due to a positive PCR for CMV DNA in amniotic fluid and women without fetal infection at the time of amniocentesis (Figure 3). Women who started VCV immediately after the diagnosis (Group A) reached the threshold for immune maturation against CMV in 18.22 (±1.21) weeks, while the patients taking the therapy after at least 6–8 weeks from the infection and presenting asymptomatic fetal infection (Group B) showed a more rapid increase, reaching a high avidity in 14.52 (±1.64) weeks (mean difference 3.7 weeks, *p* = 0.066).

## 4. Discussion

In this study, we focused on testing the potential effects of prolonged high-dose VCV administration on the progression of maternal immunological adaptation. To this end, we compared the results from a population of women with primary infection and the presence of CMV DNA in amniotic fluid who underwent 8 gr/day VCV from the confirmation of the diagnosis to the delivery with a group of women in whom VCV was started only after positive amniocentesis.

The longitudinal serological evaluation of pregnant patients presenting primary CMV infection and treated with VCV in case of fetal infection provided very significant evidence of the potential effect of this drug on maternal immune response maturation. Firstly, we found no relevant differences in the IgM and IgG trends during pregnancy, confirming that no influence is expected from VCV administration (Figure 1). Comparing these trends with those reported in the literature showed no considerable differences from the expected results [18,21]. The most significant aspect concerns the longitudinal trends of IgG avidity for CMV, the indirect sign of maternal immunological response development. Avidity maturation refers to the process by which the affinity and strength of antibody-antigen binding increase over time, typically due to somatic hypermutation and clonal selection of B cells in the germinal centers of lymphoid tissues [27]. High avidity means that the specific antibody binds more tightly to the CMV antigen, reflecting a mature and well-developed immune response. It has been suggested that antiviral drugs, such as VCV, could impact the process of avidity maturation [28]. The efficacy of VCV oral administration depends entirely on its rapid hydrolysis to Acyclovir, which is then phosphorylated by a protein kinase (encoded by the UL97 gene) to Acyclovir monophosphate. This compound accumulates in herpesvirus-infected cells, selectively inhibiting/inactivating viral DNA polymerase and viral replication. While VCV is largely recommended in pregnancy, Ganciclovir (and its prodrug Valganciclovir), a nucleoside analog of guanosine, is primarily used to treat CMV infections in adults and newborns. It is a prodrug that requires activation by phosphorylation to its triphosphate form. In this way, it competes with deoxyguanosine triphosphate for incorporation into the viral DNA chain by the CMV DNA polymerase. Once incorporated, it acts as a chain terminator, inhibiting further viral DNA synthesis. Hamilton et al. proved ex vivo the superiority of Ganciclovir over Acyclovir in CMV treatment [29]. Despite its extensive use in CMV infection treatment (particularly in high-risk populations), Ganciclovir is avoided during pregnancy due to its teratogenic and embryotoxic potential effects on the fetus and the availability of safer alternatives [30].

A peculiar aspect should be considered in evaluating the trends of affinity maturation in our CMV-infected population. Considering the overall population of the study (Figure 2), the increase in IgG avidity is consistent with the results reported by Prince et al. with a mean time-to-reach high avidity of 15.02 weeks (3.7 months from infection). Very interestingly, the avidity of the patients who started the therapy significantly earlier reached a value considered high later than the one in the group of patients who started VCV after positive amniocentesis (mean difference 3.7 weeks). As shown in Figure 3, the increase in avidity is less pronounced in Group A (VCV therapy since the diagnosis), and the values remain consistently lower compared to Group B.

Several hypotheses and speculations can explain this phenomenon. Antiviral drugs might influence the process of avidity maturation in several ways, such as altering the antigen presentation, modifying cytokine signaling, directly affecting the immune system, or, more specifically, B cell function. VCV does not directly target B cells unless they are actively infected by a herpesvirus, and this is not the case with CMV infection. While VCV does not directly target the process of avidity maturation, it can influence it by reducing the viral load and, in doing so, decreasing the amount of antigen present. By suppressing viral replication, the quantity of viral antigens available for presentation by antigen-presenting cells (APCs) like dendritic cells, macrophages, and B cells can be diminished. A reduced viral antigen burden may decrease the activation of T cells and potentially alter the immune response. A lower quantity of circulating viral antigen traduces into a reduced antigenic stimulation on the immune system. This could potentially affect the intensity and duration of the immune stimulation, which might alter the process of avidity maturation. The results of our study suggest that even though the overall IgG avidity is lower in the patients undergoing VCV therapy during pregnancy, the time to reach the high affinity for the virus remains almost the same as expected (between 12 and 16 weeks from the infection).

The effects of drugs on the avidity maturation process in the case of infection from Toxoplasma Gondii undergoing Spiramycin were already proposed by Meroni et al. in 2009 [31]. The authors found that treated pregnant women developed a lower total IgG antibody compared to a non-pregnant untreated population. Moreover, T. gondii-specific IgG avidity increased slower than expected, probably due to a reduced parasite load as suggested by the authors.

The main limitations of this single-center study are its retrospective nature, the lack of an untreated control group, and the difference in numerosity between the two groups considered. Other aspects that could lower the impact of our results are longitudinal follow-up restrictions, such as the lack of neonatal outcomes, and the basic statistical analysis that can be performed to evaluate the trend in IgG avidity since the confirmation of the diagnosis of CMV infection. Furthermore, this study did not evaluate CD4 and CD8 T cells. Without assessing these cells, we cannot determine whether the IgG avidity maturation is linked to adequate T-cell help or an effective cytotoxic response. Future studies should include CD4 and CD8 T cell assessments to measure CMV-specific T cell responses (e.g., cytokine production, proliferation, cytotoxicity) and to correlate T cell functionality with IgG avidity indices.

As far as we know, this study is the first to assess the long-term trend of IgG avidity after primary CMV infection during pregnancy. It is particularly distinctive because our research focused solely on patients with positive amniocentesis and fetuses who were asymptomatic throughout the pregnancy.

## 5. Conclusions

In summary, while high-dose VCV does not directly target the mechanism of IgG avidity maturation, it can interfere with this process by reducing the viral load and antigen presentation, influencing IgG avidity maturation. VCV’s effects on IgG avidity illustrate how antiviral therapies can potentially condition the natural course of immune response development during primary infections without altering the immunological adaptation. Our findings suggest that the antiviral therapy seems to slow the avidity increase in the group of patients who started the therapy earlier. But a very important consideration should be taken into account. While high avidity reflects a more mature antibody response, it does not directly correlate with overall immune competence. In fact, the IgG avidity value indicates the time since the primary infection, not the current immune response efficiency against the CMV infection. Very interestingly, during treatment with high-dose VCV, CMV remains in a “latent state” in certain cells, unaffected by the drug because of the absence of active viral replication. While VCV can help control viral reactivation and reduce disease symptoms, it does not eliminate the latent virus, meaning CMV can still potentially reactivate in the future under specific conditions. This underlines the importance of treatment prosecution during pregnancy to increase the probability of viral latency and non-reactivation.

Further studies are needed to ascertain those specific conditions in which the CMV can reactivate after a viral latency induced by anti-viral therapy. Since VCV does not eliminate latent CMV, long-term treatment may be necessary in patients at a high risk of reactivation. Stopping treatment or gaps in dosing can lead to reactivation from these latent reservoirs, as the virus can resume active replication when immune control is reduced. These results add precious information about VCV effects on CMV infection, encouraging antiviral therapy administration in the case of primary infection in pregnancy, even in the case of no signs of fetal as in amniotic fluid negative for CMV genome.

A thorough cost-benefit analysis is essential for implementing universal therapy with VCV for CMV infection during pregnancy due to its significant public health implications. Benefits include reduced maternal–fetal CMV transmission, fewer symptomatic cases, and improved neonatal health outcomes, potentially leading to long-term cost savings from decreased special education and medical care needs.

Key expenses involve extensive CMV testing, drug costs, and necessary monitoring and follow-up. Additionally, treating women who may not benefit, particularly in high seroprevalence areas, raises concerns about unnecessary costs and potential side effects of valacyclovir (although rare). Ethical and equity issues also arise, requiring solutions for healthcare disparities, especially in low-resource settings. Targeting high-risk groups and conducting pilot studies in high-prevalence areas could provide valuable insights into feasibility and cost effectiveness. While universal therapy may yield significant health benefits, carefully considering its logistical, ethical, and clinical impacts is crucial.

## Figures and Tables

**Table 1:** Characteristics of the population.

Characteristics	Group A N = 8	Group B N = 30	*p*-Value
Maternal Age (years)	30.25 ± 4.71	31.13 ± 4.4	0.643
Parity			
0	2 (25.0)	13 (43.3)	0.592
1	5 (62.5)	16 (53.3)	0.949
>1	1 (12.5)	1 (3.3)	0.888
Symptoms: Fever (n)	1 (12.5)	6 (20.0)	0.978
Trimester of infection			
Periconceptional	1 (12.5)	10 (33.3)	0.474
First trimester	7 (87.5)	15 (50.0)	0.132
Second trimester	0 (0)	5 (16.7)	0.515
GA at diagnosis (weeks)	10.37 ± 3.81	8.7 ± 4.85	0.317
Virus localization at diagnosis			
Blood only	1 (12.5)	4 (13.3)	0.598
Saliva only	0 (0)	2 (6.7)	0.888
Urine only	2 (25.0)	3 (10.0)	0.598
Blood + Saliva	1 (12.5)	2 (6.7)	0.846
Blood + Urine	0 (0)	2 (6.7)	0.888
Urine + Saliva	2 (25.0)	4 (13.3)	0.796
Blood + Saliva + Urine	1 (12.5)	11 (36.7)	0.379
Negative	1 (12.5)	2 (6.7)	0.846
Initiation of therapy (weeks)	14.62 ± 2.72	22.2 ± 1.69	<0.001
GA at amniocentesis (weeks)	21.75 ± 1.38	21.33 ± 1.98	0.505
Viral load on amniotic fluid (millions of copies/mL)	6.35 ± 1.02	7.71 ± 2.36	0.113
Weeks of therapy (n)	24.00 ± 3.38	17.26 ± 2.44	<0.001

GA gestational age.

## Data Availability

The raw data supporting the conclusions of this article will be made available by the authors upon request.
